# Risk factors of loss of reduction after acromioclavicular joint dislocation treated with a hook plate

**DOI:** 10.1186/s10195-023-00685-8

**Published:** 2023-03-24

**Authors:** Young Seok Lee, Doo Sup Kim, Ji Won Jung, Young-Hoon Jo, Chang-Hun Lee, Bong Gun Lee

**Affiliations:** 1 Department of Orthopedic Surgery, Hanyang University Guri Hospital, Guri, Republic of Korea; 2 Department of Orthopedic Surgery, Yonsei University Wonju College of Medicine, Wonju, Republic of Korea; 3 Department of Orthopedic Surgery, College of Medicine, Hanyang University, Seoul, Republic of Korea

**Keywords:** Acromioclavicular joint injury, Hook plate, Dislocation, Loss of reduction, Comparison, Outcomes

## Abstract

**Background:**

Acromioclavicular joint fixation using a hook plate is effective for the treatment of acute acromioclavicular joint dislocation. However, several studies have reported some complications including loss of reduction after surgery for acromioclavicular joint dislocation. This study aimed to identify the risk factors associated with the loss of reduction after acromioclavicular joint dislocation surgery using a hook plate.

**Methods:**

This was a retrospective study that assessed 118 patients with acromioclavicular joint dislocation, who were diagnosed between March 2013 and January 2019 and underwent surgical treatment using the hook plate (reduction loss group: *n* = 38; maintenance group: *n* = 80). The mean follow-up period was 29.9 months (range, 24–40 months). We assessed the range of motion, the American Shoulder and Elbow Surgeons score (ASES), visual analog scale score for pain, and a subjective shoulder value. Radiological assessment of coracoid clavicular distance was performed. The risk factors of reduction loss were analyzed using multivariable logistic regression analysis.

**Results:**

Age (*p* = 0.049), sex (female, *p* = 0.03, odds ratio OR = 4.81), Rockwood type V (*p* = 0.049, OR = 2.20), and time from injury to surgery > 7 days (*p* = 0.018, OR = 2.59) were statistically significant factors in the reduction loss group. There were no significant differences in the clinical outcomes for range of motion, ASES, subjective shoulder value, and visual analog scale scores between the two groups. In the radiological results, preoperative coracoid clavicular distance (*p* = 0.039) and ratio (*p* = 0.001), and over-reduction (*p* = 0.023, OR = 0.40) were significantly different between the two groups. The multivariate logistic regression analysis identified the female sex (*p* = 0.037, OR = 5.88), a time from injury to surgery > 7 days (*p* = 0.019, OR = 3.36), and the preoperative coracoid clavicular displacement ratio of the injured shoulder (*p* < 0.001, OR = 1.03) as risk factors associated with reduction loss following surgery using a hook plate for acromioclavicular dislocation.

**Conclusion:**

A delayed timing of surgery > 7 days, preoperative coracoid clavicular displacement ratio of the injured shoulder, and female sex were identified as risk factors for loss of reduction after surgery using a hook plate for acromioclavicular joint dislocation.

*Level of evidence*: Level IV; retrospective comparison; treatment study

## Introduction

Acromioclavicular (AC) joint injury is a common injury of the shoulder, accounting for 9–12% of all shoulder injuries [1]. The surgical method is commonly determined on the basis of the Rockwood classification, which proposed six types of injury in 1984, and is the most widely used classification system for AC joint injury [2]. In Rockwood types III and V, the AC ligaments and coracoclavicular (CC) ligaments are completely disrupted, which results in vertical instability and an increased CC interval. Type III injuries are defined as having CC intervals widened up to 100% compared with the CC intervals of the contralateral side; type V are injuries that have widening of the CC intervals from 100% to 300%. Surgical treatment is required for types IV–VI, and conservative versus surgical treatment decisions are controversial for type III injuries. The currently available data for Rockwood type III injuries is insufficient to support any treatment [3–5].

Several studies have shown that surgical treatment for AC joint injury is appropriate for patients with high physical needs, such as young people, athletes, and physical workers [6–8]. Various surgical options have been developed to manage AC joint dislocation, including coracoacromial ligament transfer (Weaver–Dunn procedure), CC fixation, and AC or CC reconstruction [9, 10]. Among the methods of AC joint fixation, using a hook plate is effective for the treatment of acute AC joint dislocation and has the advantage of being a simple technique that allows early joint motion due to high stability [11–13]. However, the implant generally needs to be removed early to avoid subacromial osteolysis and AC joint arthrosis [14]. Other complications have been reported, including hook plate displacement from the subacromial space, migration into the acromion, and clavicle fracture [7, 15–17]. The most common complication is loss of reduction, with incidence rates of 15–80% in previous studies [15, 18–21].

Several studies have identified risk factors of loss of reduction after surgery for AC joint dislocation. Sun et al. [22] reported that osteoporosis, clavicle tunnel position, and the position of the coracoid process button were statistically significant risk factors for loss of reduction after AC joint dislocation surgery that used a suture-button technique. However, no studies have investigated the risk factors that affect the loss of reduction after surgery that uses a hook plate for AC joint dislocation. Therefore, this study analyzed postsurgical patient-related outcomes to identify risk factors associated with the loss of reduction following AC joint dislocation surgery using a hook plate, with an aim toward how it might be prevented. Our hypothesis is that early operative treatment within 7 days would effectively prevent reduction loss after AC joint dislocation surgery using a hook plate.

## Methods

### Patients

This was a retrospective study of patients with AC joint dislocation who underwent surgical treatment using the AO hook plate (clavicular hook plate, Synthes, Switzerland) at three general hospitals between March 2013 and January 2019. AC joint dislocations were diagnosed by orthopedic surgeons following clinical and radiological evaluations. The patients were assigned into two groups based on AC joint re-dislocation (reduction loss group and maintenance group). The inclusion criteria were a Rockwood classification of type III or V, acute injury within 6 weeks after the initial trauma, and at least 2 years of follow-up with clinical and radiological evaluations. The exclusion criteria were chronic injuries for > 6 weeks after the initial trauma, a previous operation history for an injured shoulder joint, a combined neurovascular injury, and a combined fracture of the upper extremities on the injured side. This study was approved by our institutional review board (HYUH-2020-06-037).

### Surgical technique

All the surgeries were performed under general anesthesia, with the patient in a beach chair position and the injured limb freely mobile. A transverse 5–6 cm incision was made above the distal clavicle and AC joint. The deltoid–trapezoidal fascia was incised in line with the lateral clavicle. After exposure, temporary reduction of the AC joint was achieved using a 1 or 2 K-wire fixation. Then, a hook plate was placed under the acromion, posterior to the AC joint, and fixed with a 3.5 mm medial cortical screw. Fluoroscopic imaging was used to observe the reduction status, depth of the hook, and contour of the plate on the distal clavicle. After confirming the proper position of the hook plate, locking screws were inserted in all holes. The ruptured AC ligament was repaired after fixation of the hook plate only when the remnant ligament was enough to repair, using the trans-osseous suture technique by one of the three surgeons. The surgeon passed suture material through the distal part of the clavicle and sutured the remnant tissue around the AC joint. The other two surgeons did not participate in the AC ligament repair. Stable repair of the delto–trapezial fascia was done.

All patients initiated passive range of motion exercise immediately after the surgery and were immobilized with an arm sling for 2 weeks. Progressive rehabilitation was conducted for all patients under the operator’s direction, and they were allowed to use their affected arm for daily activities 6 weeks after the surgery. The hook plate was removed routinely 3−4 months after the initial surgery.

### Clinical assessment

Demographic and clinical data were reviewed using the patients’ electronic medical records. The demographic data included age, sex, body mass index (BMI), injured side, injury mechanism, Rockwood classification, time from injury to surgery, whether AC ligaments were repaired or not, and time from internal fixation to implant removal. The clinical outcomes were assessed using the American Shoulder and Elbow Surgeons (ASES) score, visual analog scale (VAS) score for pain, and subjective shoulder value (SSV) when a patient visited the hospital for the last follow-up. The overall satisfaction with surgery was quantified with a maximum score of 100 points. Shoulder joint range of motion (ROM) was measured using a goniometer while the patient sat on a chair during an outpatient visit. The three general hospitals (A, B, C) were compared separately based on maintenance and reduction loss groups.

### Radiographic assessment

Radiological assessment was performed preoperatively, postoperatively, and at final follow-up using both clavicle anteroposterior plain radiographs. The images were analyzed and standardized for the assessment of the CC distance on both sides by comparison with that of the contralateral shoulder using the distance between the upper border of the coracoid process and the inferior cortex of the clavicle. The displacement ratio of the AC dislocation was defined as the ratio of the CC distance of the injured side to that of the contralateral side. Radiographs were measured using the measurement tool in a picture archiving and communication system.

$$\frac{\mathrm{CC}\, \mathrm{distance\, of\, the\, injured\, shoulder}-\mathrm{CC}\, \mathrm{distance\, of\, the\, contralateral\, shoulder\, }(\mathrm{non}-\mathrm{injured})}{\mathrm{CC}\, \mathrm{distance\, of\, the\, contralateral\, shoulder }\,(\mathrm{non}-\mathrm{injured})}\times 100 (\%)$$

**Fig. 1:**
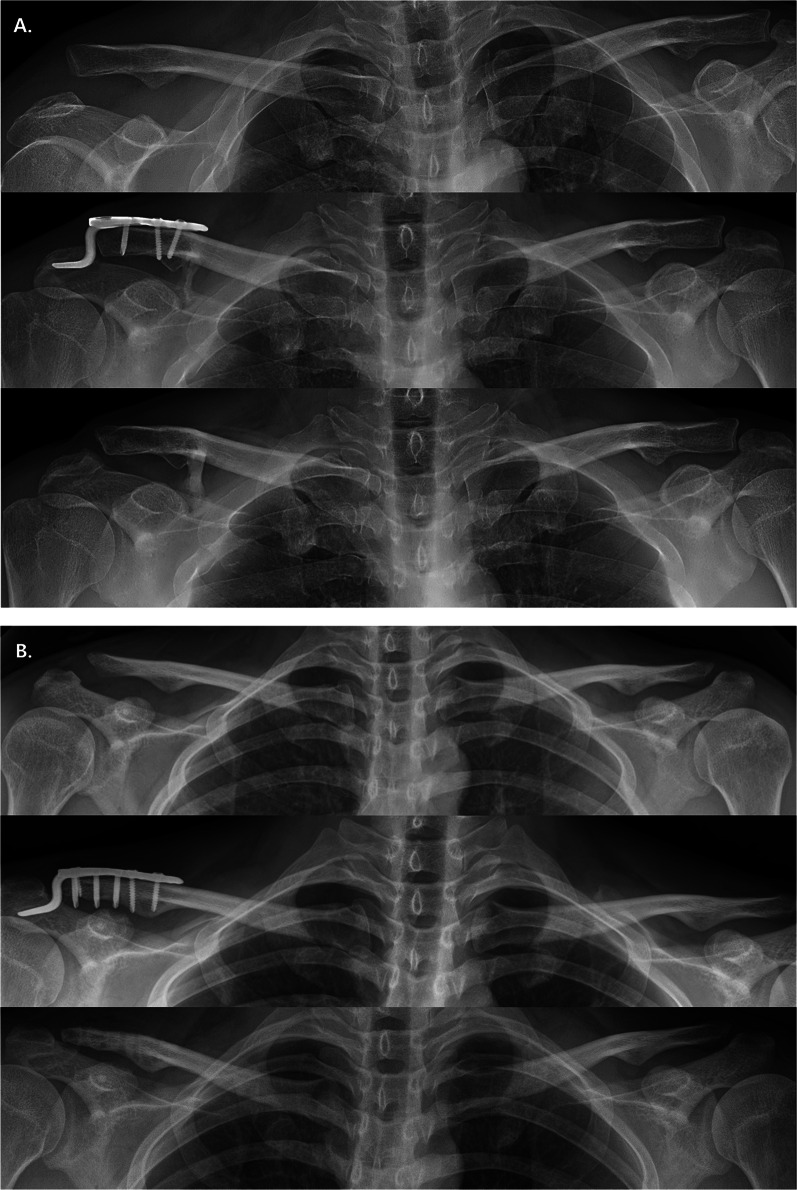
Antero-posterior radiograph of patient of AC joint injury treated by AO hook plate. **A** Reduction loss group. **B** Maintenance group

We defined a re-dislocation to be a ≥ 50% increase in the CC distance as compared with that of the contralateral side in the final follow-up radiograph [23]. Over-reduction was defined as the CC distance of the injured side being shorter than that of the contralateral side on plain radiography performed immediately after surgery. In accordance with this definition, the patients with re-dislocation were classified as the reduction loss group, and the patients without re-dislocation were classified as the maintenance group (Fig. 1). The presence of osteoarthritis was confirmed in the last follow-up plain radiography.

### Statistical analysis

For statistical analysis, SPSS ver. 22.0 (SPSS Inc., Chicago, IL, USA) was used, and a *p* < 0.05 was considered statistically significant. For continuous variables, an independent Student’s *t*-test was performed to compare the average between the two groups; Chi-square and Fisher’s exact tests were performed to analyze categorical variables. The risk factors for re-dislocation were analyzed using multivariable logistic regression analysis, and variables were selected using stepwise regression.

## Results

A total of 118 patients that met the inclusion criteria were evaluated; 80 patients and 38 patients were included in the maintenance and reduction loss groups, respectively. The demographic data of both groups are presented in Table 1, and the clinical outcomes are presented in Table 2. The mean follow-up period was 29.9 months (range, 24–40 months), and the mean age of the patients was 45.8 years (range, 15–76 years). We assessed 109 men and 9 women in the 7-year study period. The most common causes for AC dislocation injury were traffic accidents (*n* = 66, 50.8%), slipping down (*n* = 40, 33.9%), and falls (*n* = 3, 2.5%).

**Table 1:** Comparison of risk factors between the maintenance and reduction loss groups

Risk factor		Maintenance group(*n* = 80)	Reduction loss group (*n* = 38)	Total	*p*-Value
Age (years)		44.0 ± 13.8	49.5 ± 13.9	45.8 ± 14.0	0.049
Sex	Male	77 (96.3%)	32(84.2%)	109 (92.4%)	0.030
	Female	3 (3.8%)	6 (15.8%)	9 (7.6%)	
BMI (kg/m^2^)		24.9 ± 2.6	25.1 ± 2.9	25.0 ± 2.7	0.618
Cause	Bicycle accident	28 (35.0%)	6 (15.8%)	34 (28.8%)	0.113
	Motorcycle accident	3 (3.8%)	4 (10.5%)	7 (5.9%)	
	Pedestrian accident	6 (7.5%)	2 (5.3%)	8 (6.8%)	
	In-car accident	9 (11.3%)	8 (21.1%)	17 (14.4%)	
	Slip down	28 (35.0%)	12 (31.6%)	40 (33.9%)	
	Fall down	4 (5.0%)	5 (13.2%)	9 (7.6%)	
	Other	2 (2.5%)	1 (2.6%)	3 (2.5%)	
Follow-up period (months)		28.3 ± 4.2	32.3 ± 3.7	29.9 ± 4.1	0.301
Time from injury to surgery (days)	Less than seven	56 (70.0%)	18 (47.4%)	74 (62.7%)	0.018
	More than seven	24 (30.0%)	20 (52.6%)	44 (37.3%)	
Repair of the AC ligament	Not repair	52 (65.0%)	24 (63.2%)	76 (64.4%)	0.845
	Repair	28 (35.0%)	14 (36.8%)	42 (35.6%)	
Time to implant removal (days)		130.4 ± 57.5	110.7 ± 44.5	124.1 ± 54.2	0.064
Hospital	A B C	28 (23.7%)	14 (11.9%)	42 (35.6%)	0.978
		19 (16.1%)	9 (7.6%)	28 (23.7%)	
		33 (28.0%)	15 (12.7%)	48 (40.7%)	

Data are presented as number (percentage) or mean ± SD

**Table 2:** Comparison of clinical outcomes between the maintenance and reduction loss groups

		Maintenance group (*n* = 80)	Reduction loss group (*n* = 38)	Total	*p*-Value
Postoperative	FF	173.1 ± 9.8	175.8 ± 7.2	174.0 ± 9.6	0.099
	Abd	174.6 ± 9.1	171.3 ± 9.9	173.6 ± 9.5	0.076
	ER	87.2 ± 13.2	78.8 ± 13.4	81.1 ± 13.3	0.199
	ASES	93.9 ± 5.3	94.6 ± 5.6	94.1 ± 5.4	0.211
	SSV	90.6 ± 6.2	89.0 ± 7.1	90.0 ± 6.5	0.534
VAS score	Preoperative	7.3 ± 1.2	7.3 ± 1.2	7.3 ± 1.2	0.959
	Postoperative	0.9 ± 1.0	0.8 ± 1.0	0.8 ± 1.0	0.609
Satisfaction		85.6 ± 10.8	82.6 ± 12.9	84.6 ± 11.6	0.199

Data are presented as number (percentage) or mean ± SD

*FF* forward flexion, *Abd* abduction, *ER* external rotation, *ASES* American shoulder and elbow surgeons, *SSV* shoulder subjective value, *VAS* visual analog scale for pain

The demographic data showed that significant factors in the reduction loss group were age (old age, *p* = 0.049), sex (female, *p* = 0.03; odds ratio OR = 4.81), Rockwood type V (*p* = 0.049, OR = 2.20), and a time from injury to surgery > 7 days (*p* = 0.018, OR = 2.59; 6.4 ± 6.9 versus 8.2 ± 8.1 days). No significant differences were found between the groups in BMI, injury side, injury mechanism, follow-up period, time from internal fixation to implant removal, repair of the AC ligament, or different hospitals. We found no significant differences in clinical outcomes for ROM, ASES, SSV, VAS score, or satisfaction score between the two groups. In the radiological results, significant differences between the two groups were identified for preoperative CC distance (*p* = 0.039), ratio (*p* = 0.001), and over-reduction (*p* = 0.023, OR = 0.40) (Table 3).

**Table 3:** Comparison of radiographic factors between the maintenance and reduction loss groups

		Maintenance group (*n* = 80)	Reduction loss group (*n* = 38)	Total	*p*-Value
CC distance of the injured shoulder(mm)	Preoperative	16.3 ± 4.8	18.3 ± 5.1	17.0 ± 4.9	0.039
	Postoperative	7.2 ± 3.3	8.2 ± 3.1	7.5 ± 3.2	0.147
CC displacement ratio of the injured shoulder (%)	Preoperative	92.8 ± 51.1	129.6 ± 68.0	104.6 ± 59.4	0.004
	Postoperative	−17.8 ± 28.3	2.1 ± 32.4	−11.4 ± 31.0	0.001
Over-reduction	No over-reduction (≥ 0%)	21 (26.3%)	18 (47.4%)	39 (33.1%)	0.023
	Over-reduction (< 0%)	59 (73.8%)	20 (52.6%)	79 (66.9%)	
Rockwood classification	III	45 (56.3%)	14 (36.8%)	59 (50%)	0.049
	V	35 (43.8%)	24 (63.2%)	59 (50%)	

Data are presented as number (percentage) or mean ± SD

The data of the over-reduction group was further analyzed. In the last follow-up, 21.1% of the X-rays showed OA changes, but there was no statistically significant difference between the group with or without over-reduction. Even when OA changes were shown, there was no significant effect in clinical outcomes for ASES or satisfaction score. (Table 4).

**Table 4:** Comparison of radiographic and clinical factors between the osteoarthritis and non-osteoarthritis groups

		Osteoarthritis group (*n* = 25)	Non-osteoarthritis group (*n* = 93)	Total	*p*-Value
Reduction maintenance	Maintenance group	16 (20.0%)	64 (80.0%)	80 (67.8%)	
	Reduction loss group	9 (23.6%)	29 (76.4%)	38 (32.2%)	0.647
Over-reduction	No over-reduction (≥ 0%)	8 (20.5%)	31 (79.5%)	39 (33.1%)	0.900
	Over-reduction (< 0%)	17 (21.5%)	62 (78.5%)	79 (66.9%)	
ASES		92.2 ± 5.3	94.6 ± 5.6	94.1 ± 5.4	0.321
Satisfaction		80.4 ± 10.8	85.7 ± 10.8	84.6 ± 11.6	0.229

Multivariate logistic regression analysis (Table 5) identified the female sex (*p* = 0.037, OR = 5.88), time from injury to surgery > 7 days (*p* = 0.019, OR = 3.36), and preoperative CC displacement ratio of the injured shoulder (%) (*p* < 0.001, OR = 1.03) as risk factors associated with re-dislocation in patients treated with surgery using a hook plate for AC dislocation.

**Table 5:** Results of the multivariate logistic regression analysis of the effective risk factors

Risk factor	Odds ratio	95% CI	*p*-Value
Sex	5.88	4.21–7.55	0.037
Time from injury to surgery	3.36	2.34–4.38	0.019
Preoperative CC displacement ratio of injured shoulder (%)	1.03	1.01–1.05	< 0.001

## Discussion

AC joint injury occurs mainly in young people aged 37.5 years (range, 13–69 years), and sports injuries are the most common injuries [24]. The mean age of the patients (45.8 years) enrolled in this study was higher than that of the previous study, and the injury mechanisms in this study have also differed from those reported in the previous study. In this study, the most common injury mechanism was traffic accidents (55.9%), followed by slipping down (33.9%) and falls (7.6%). This is because our study includes only Rockwood type III and V, and young patients with Rockwood type I or II, injured by a sports injury may have been excluded from this study.

The mean age was significantly different between the reduction loss and maintenance groups, with the reduction loss group having significantly older patients. According to Nakano et al. [25], younger patients had more healing potential than older patients in a study of the association between injured ligament and age. Similarly, the present study also showed a correlation between age and the healing potential of an injured ligament.

The proportion of women in the reduction loss group was significantly higher than that in the maintenance group. In addition, multivariate logistic regression analysis showed that the female sex was a significant risk factor of loss of reduction. This difference can be attributed to men and women having different muscle mass and ligamentous laxity. In addition, women have higher circulating relaxin hormone, which influences ligament laxity; relaxin diminishes ligament integrity, which has been shown to act as a risk factor for ACL tear and shoulder instability in previous studies [26].

According to a systematic review [27], early surgical treatment within 3 weeks for AC joint dislocation has better clinical and radiological outcomes; therefore, we tried to perform surgery early. The patients in this study underwent surgery 6.97 days after injury; based on this average, we classified them into subgroups of more > 7 days and < 7 days. The delayed surgery group (> 7 days after injury) had a significantly higher incidence rate of reduction loss than the early group (< 7 days after injury). In addition, multivariate logistic regression analysis confirmed that the time from injury to surgery was a significant risk factor of loss of reduction. Cook et al. [28] reported that patients with chronic AC dislocation (> 2 months) had worse outcomes than those with acute AC dislocation (< 2 weeks) following surgical treatments for AC joint dislocation. According to Weinstein et al. [20], if surgery is performed 3 weeks after AC dislocation, the healing potential is lost; therefore, they suggested that surgery must be performed within 3 weeks after injury. In a systematic review, Song et al. [27] reported that patients who had an early surgery had better Constant scores and overall outcomes than those who had a delayed surgery. In our clinical experience, the surgeries for AC joint injury were often delayed by more than 7 days due to patients’ other conditions, such as comorbidities or associated injuries. Based on the present study, it is recommended that surgery for AC joint dislocation be performed in < 7 days, if possible.

When comparing the radiological factors, we found that the preoperative CC distance and displacement ratio in the reduction loss group were significantly greater than in the maintenance group. This means that the proportion of patients with Rockwood classification type V in the reduction loss group was significantly higher, as shown in the statistical analysis. Moreover, the multivariate logistic regression analysis revealed that the greater the CC displacement ratio before surgery, the greater the risk of reduction loss after hook plate removal. Although the clinical implication may be minimal compared with other factors with an odds ratio of 1.03 (*p* < 0.01), it still proves correlation with reduction loss. In Rockwood type V injuries, damage to the soft tissue around the AC joint and ligament is more extensive than in type III. Therefore, the damage to the structure that affects the stability of the AC joint may be more significant in type V. This is thought to be the main reason for the greater rate of reduction loss in type V injuries. In their biomechanical study, Hislop et al. [29] reported that the stability of the AC joint affects not only the AC and CC ligaments, but also the soft tissues around the AC joint, such as the AC joint capsule and delto–trapezial fascia.

When comparing the postoperative CC displacement ratio, we found that the surgical reduction was more excessive in the maintenance group than in the reduction loss group. The proportion of patients with over-reduction after surgery was statistically higher in the maintenance group than in the reduction loss group. Therefore, over-reduction during surgery with an AO hook plate for AC joint dislocation could be a factor for preventing reduction loss.

In this study, the mean time from initial surgery to implant removal differed by around 20 days between the two groups, but the difference was not statistically significant (*p* = 0.064). Due to the nature of the retrospective review, not all causes of rapid removal were recorded in the medical record, but many complained of discomfort related to the implant, such as limited shoulder motion and shoulder pain. It is known from previous studies[16, 30] that the AO hook plate should be removed 3 months after surgery to prevent subacromial impingement. However, we are concerned about reduction loss after early implant removal. We removed the implant at approximately the same time as in previous studies, but the implant was removed around 20 days earlier in the reduction loss group than in the maintenance group. Therefore, additional studies are needed to clarify the relationship between the timing of implant removal, the incidence of reduction loss, and subacromial impingement.

The effectiveness of AC ligament repair after reduction in patients with AC dislocation is controversial [7, 15, 16]. According to a systematic review by Jordan et al. [31], additional AC ligament repair after CC stabilization showed better results in biomechanical studies than CC stabilization alone, and they reported significant differences in clinical results such as ASES and Constant scores. However, by comparing the reduction loss and maintenance groups in the present study, we found that AC ligament repair did not affect the reduction loss (*p* = 0.845). Therefore, further biomechanical and long-term follow-up studies are needed to elucidate the effect of AC ligament repair performed after fixation using AO hook plates.

The ROM and clinical outcomes measured at the last follow-up were not significantly different between the two groups. Therefore, we concluded that reduction loss did not have a significant effect on the clinical results during the short-term follow-up. The clinical outcomes showed no significant differences between the two groups because the scoring systems that were used did not sufficiently reflect the function of the AC joint, owing to the ceiling effect reported in the study by Lee et al. [32] In addition, symptoms are often mild, even if reduction loss occurs. In this study, five patients in the reduction loss group complained of discomfort and instability of the AC joint area.

This study has some limitations. First, this was a nonrandomized retrospective study. Second, as mentioned above, due to the characteristics of traumatic injury, the number of women (*n* = 9, 7.6%) enrolled in this study was smaller than the number of men (*n* = 109, 92.4%) because the incidence in women is low. Further studies on a larger number of AC joint ligament injury in the female population is required to find potential sex-related factors that may affect the results.

Third, the clinical and radiologic parameters were measured once by each observer and thus, intraobserver variability could not be evaluated. Fourth, we did not evaluate the horizontal instability of the AC joint. The AC joint dislocation leads to vertical and horizontal instability of the AC joint. Horizontal instability is difficult to quantify with the use of standard radiographs. The axillary shoulder X-ray view is valuable in evaluating horizontal instability. But we did not check the axillary view routinely. In this study, there was no statistically significant relationship between AC ligament repair with re-dislocation or clinical outcome. However, if the effects of AC joint repair or augmentation on horizontal stability can be evaluated, the understanding of AC joint dislocation could improve. Lastly, the patients enrolled in this study underwent surgeries that three surgeons in three hospitals performed; therefore, the surgical method, postoperative care, and rehabilitation process could have differences. However, the results showed no significant difference and because the surgical procedure is relatively simple, and the general principles of postoperative care were very similar between the hospitals, the study could be generalized to other patients. Despite these limitations, this is the first study to evaluate the risk factors of loss of reduction after using a hook plate in AC joint dislocation surgery.

## Conclusion

This study confirmed our hypothesis that delayed timing of surgery of > 7 days was a risk factor for loss of reduction after surgery using a hook plate for AC joint dislocation. In addition, it also identified the preoperative CC displacement ratio of the injured shoulder and the female sex as risk factors.

## Data Availability

The datasets during and/or analyzed during the current study are available from the corresponding author on reasonable request.
